# Changes in family income status and the development of overweight and obesity from 2 to 15 years: a longitudinal study

**DOI:** 10.1186/1471-2458-14-417

**Published:** 2014-05-01

**Authors:** Margaret M Demment, Jere D Haas, Christine M Olson

**Affiliations:** 1 Division of Nutritional Sciences, Cornell University, 352 Martha Van Rensselaer Hall, Ithaca, NY, USA

**Keywords:** BMI trajectory, Low-income trajectory, Latent-class modeling, Obesity, Longitudinal, Lifecourse

## Abstract

**Background:**

An emerging body of research suggests the trajectory of a family’s income affects children’s health and development more profoundly than the often-measured income at a single time point. The purpose of this study was to examine the associations between changes in family income status, early-life risk factors, and body mass index (BMI) z-score trajectory from age 2 to 15 years.

**Methods:**

This longitudinal study employed a birth cohort (n = 595) located in a rural region of New York State. Data were collected through an audit of medical records and mailed questionnaires. Family low-income and BMI z-score trajectories were identified using latent-class modeling techniques that group children based on similar trends across time. We examined five early-life risk factors in relation to income and BMI z-score trajectories: maternal overweight/obesity, maternal gestational weight gain, maternal smoking during pregnancy, breastfeeding duration, and early-life weight gain trajectory. We used multinomial logistic regression models to estimate the odds of being in a BMI z-score trajectory group based on income trajectory and early-life risk factors.

**Results:**

Children who remain low-income throughout childhood were more likely to maintain overweight (AOR = 2.55, 95% CI = 1.03, 5.42) and children who moved into low-income during childhood were more likely to be obese (AOR = 2.36, 95% CI = 1.12, 5.93) compared to children who were never low-income. Maternal overweight/obesity was significantly associated with a child become obese (AOR = 8.31, 95% CI = 3.80, 18.20), become overweight (AOR = 2.37, 95% CI = 1.34, 4.22), and stay overweight (AOR = 1.79, 95% CI = 1.02, 3.14). Excessive gestational weight gain was associated with increased likelihood of a child becoming overweight trajectory (AOR = 2.01, 95% CI = 1.01, 4.00).

**Conclusions:**

Our findings further supports the growing evidence that there are several preventable early-life risk factors that could be targeted for intervention. This study provides new evidence that remaining in low-income and moving into low-income increases risk for adolescent overweight and obesity.

## Background

Income inequalities in health are substantial in the United States and have risen over the past several decades [1]. While ample evidence suggests that poorer children do not perform as well as higher income children on a wide variety of health, occupational, and educational measures, the evidence is inconclusive on the association between family income and childhood body mass index (BMI). Most studies have examined cross-sectional trends in the association between childhood overweight and SES in the US [2,3].

One reason for the inconclusive evidence may be that cross-sectional measures of family income fail to capture critical aspects of the experience of being low-income. An emerging body of research suggests that it is the persistence and trajectory of the family’s low-income, rather than the often-measured overall level of deprivation at a single point in time, that affects children’s health and development [4-6]. Income is often only measured once and this can be problematic, especially given how dynamic income is in the United States [7]. Therefore, it is important to conceptualize the relationship of family low-income to health outcomes using the lifecourse perspective. One feature of a lifecourse perspective is trajectories, patterns of experiences, behaviors, or health across time [8]. Typically, a person’s trajectories for health, social status, and other personal characteristics develop together. Consequently, a change in one trajectory, such as family low-income, may lead to changes in other trajectories, such as body weight.

The majority of studies that have examined the relationship between childhood family income and obesity have used adult populations, cross-sectional data, and one measure of childhood SES. These studies have found that childhood SES is an important predictor of adult BMI [9-17]. There are only a handful of studies that have examined the longitudinal relationship between SES and the development of obesity from adolescence to adulthood [9,18,19]. In addition, two studies have employed a longitudinal measure of SES and single measure of weight status in adolescence [20,21]. Hallal et al. [21] found that richer adolescents were more likely to be overweight in a Brazil population, while Kendzor et al. [20], found that downward mobility and stable low income were associated with greater adiposity at age 15 relative to the more advantaged income trajectories in the US. There are currently no studies that have examined changes in income status and the development of overweight and obesity from early-life to adolescence.

Few studies have examined the relationship of early-life obesity risk factors and weight trajectories across childhood [22,23]. The developmental origins hypothesis [24] suggests that early exposure to undernutrition or overnutrition *in utero*[25], feeding practices in infancy [26-28], smoking during pregnancy [29], and weight gain in early childhood [30] may play a role in the development of obesity in adult life. We hypothesize that these early-life obesity risk factors could contribute to the development of obesity in childhood.

Guided by the lifecourse perspective, the aim of this study was to use repeated measures across time to create trajectories for both family income and BMI and then examine the association between these two variables in the context of early-life obesity risk factors. We hypothesized that children whose income trajectories included time spent in low-income are more likely to become overweight or obese. The findings of this study provide new insight into the development of overweight and obesity in children.

## Methods

### Study sample

This study used data from the Bassett Mothers Health Project (BMHP1), an observational cohort study of 622 healthy, adult women followed from early pregnancy until 2 years postpartum. Women were recruited from the population registering for prenatal care at Bassett Healthcare’s network of primary care clinics in an 8-county area of rural New York State. Additional eligibility and participation details are described elsewhere [31,32]. The population-based birth cohort for this study is the 595 full-term children born to these women from June 1995 to July 1997. The sample is predominantly white (96%) with a high proportion of children born to families who were low-income, below 185% of federal poverty limits, at the time of the birth (43%).

### Data collection

This study uses data from two sources. The first source was information gathered from mothers enrolled in the BMHP1. BMHP1 was designed to examine the biological, behavioral, psychological and sociodemographic characteristics of women and the relationships of these characteristics to postpartum weight retention [31]. Medical chart audits, mailed questionnaires, and measurement of maternal height and weight were used to collect information on the mothers. The second source of data was retrospective medical chart audits for each full-term child born to BMHP1 mothers. The aim of the medical chart audits was to collect data on the health and growth of the children. Medical chart audits were conducted between August 2009 and August 2011. The study was approved by the Institutional Review Boards of Cornell University and Bassett Healthcare.

### BMI z-score trajectory: 2 to 15 years

Measured heights and weights for each child were obtained through the medical chart audit and used to calculate BMI z-scores, using the World Health Organization 2007 growth standards [33]. The Bassett Healthcare Network has a standard protocol used by all clinics for obtaining heights and weights. Measurement frequency and timing were different for each child. Inclusion criteria for this study was at least one BMI z-score measurement after age 2, this reduced the sample to 87% of the population-based sample. Frequency of BMI z-score measurements was dictated largely by the regularly scheduled well-child exam visits, 62% of children in the analysis sample had BMI measurements every 3 years from 2 to 15 years of age. The median number of BMI z-score measurements per child in the sample was 16 (25-75% quantile: 10–21). We used maximum-likelihood longitudinal latent-class modeling techniques to identify BMI z-score trajectories and classify children.

### Family income trajectory: 0 to 15 years

Children’s income trajectories were based on their family’s movement in and out of low-income from birth to 15 years of age. Income was based on insurance codes recorded in the medical records for billing purposes at the time of a child’s visit to a clinic or hospital. Children were classified as low-income if their insurance was listed as Medicaid or Child Health Plus, which requires families to have incomes of 185% of the Federal poverty line or less. In the sample, 50% of the children had at least one income measurement every three years. The median number of income measurements per child was 12 (25-75% quantile: 6–19). We used maximum-likelihood longitudinal latent-class modeling techniques to identify family income trajectories and classify children.

### Early-life risk factors

#### Maternal overweight/obesity category

Measured heights and weights from early pregnancy were recorded from medical chart audits as part of the BMHP1 study. Maternal overweight/obesity was classified using the Institute of Medicine BMI cutoff of ≥ 25 kg/m^2^ (29). Maternal overweight/obesity is a dichotomous variable (yes/no).

#### Gestational weight gain

A detailed description of gestational weight gain methods is given elsewhere [34]. Briefly, the amount of weight gain was determined by subtracting the first measured weight in the first trimester of pregnancy from the weight at the last prenatal care visit, which was generally within one week of delivery. The 2009 Institute of Medicine’s BMI categories and gestational weight gain guidelines were used to determine gestational weight gain category [35]. Gestational weight gain is a categorical variable with three groups: excessive; inadequate; and adequate.

#### Maternal smoking during pregnancy

We derived smoking status during pregnancy from the medical chart audits conducted as part of the BMHP1 study. Maternal smoking during pregnancy is a dichotomous variable (yes/no).

#### Breastfeeding duration

Breastfeeding duration (including exclusive and partial breastfeeding) was derived from survey responses from the BMHP1 study. Women were grouped based on similarity in breastfeeding duration using maximum-likelihood longitudinal latent-class modeling techniques. Model fit statistics strongly supported the 4-group model (Additional file 1: Table S1). Breastfeeding duration is a categorical variable with four groups: <1 month; 1 to <4 months; 4 to <8 months; and ≥8 months.

#### Early-life rapid weight gain

Weight-for-length (WFL) z-scores were calculated for all height and weight data in the first two years of life (including birth weight and length) using the World Health Organization 2007 growth standards [33]. We used maximum-likelihood longitudinal latent-class modeling techniques to classify early-life rapid weight gain (Additional file 2: Table S2). In this case the recommended change in BIC model fit statistic was not used for model selection as they continued to decrease as more and more groups were added, even though the additional groups did not add any extra explanatory power. Therefore, we used non-statistical criteria to select the model that best captured the biologically significant trajectories (“stable” and “rising”). Three early-life weight gain trajectories were identified: high-rapid weight gain (35% of the sample); low-rapid weight gain (42%); and stable weight gain (23%) (Additional file 3: Figure S1). Children in the high-rapid weight gain had a WFL z-score of 0 at birth and rose to above 1 by the time they were 2 years old. Children with low-rapid weight gain trajectories had a WFL z-score of around −1 at birth and rose to around 0.5 by the time they were 2 years old. Children had stable weight gain had WFL trajectories that remained stable at about 0 WFL z-score from 0–2 years.

### Analysis

#### BMI z-score trajectory and family income trajectory

Group-based trajectories for income from 0 to 15 years and BMI z-score from 2 to 15 years were identified using maximum likelihood latent-class models in PROC TRAJ [36,37]. The purpose of this modeling was to characterize the optimal number of trajectories over time based on statistical and public health criteria. Models with 1 to 8 classes were estimated for BMI trajectory and 1 to 6 for income trajectory, beginning with the simplest model. Model fit for each of the subsequent models was first statistically evaluated based on recommended change in Bayesian Information Criterion (BIC) score [36]. In addition, we examined three other measures for model selection based on the recommendations of Henson *et al.*[38]: entropy, sample-size adjusted BIC, and integrated classification likelihood BIC. After the review of model statistics, groups were visually inspected to insure that distinctive features of the data were summarized in as parsimonious a fashion as possible and the final model was based on identifying groups of public health significance. The two main outputs from the trajectory models were the shape of each group’s trajectory and the probabilities of being in each group. The program used the latter to classify individuals into trajectory groups [39]. These classifications were then used in the analysis as categorical variables.

#### BMI z-score trajectory classification based on family income trajectory and early-life risk factors

We examined all bivariate relationships between BMI z-score trajectory, family income trajectory, and potential early-life risk factors using Chi-squared analysis (Additional file 4: Table S3). All two-way interactions were also explored in the multivariate models. We developed multivariate multinomial logistic regression models to estimate the odds of being in a certain BMI z-score trajectory based on family income trajectory and early-life risk factors. We evaluated all variables for potential mediation using PROCESS [40] and all variables with complete mediation were removed from the final model. All models included the following maternal variables as controls as they relate to early-life obesity risk factors and the child’s BMI z-score trajectories: age of mother at time of delivery (18 to <25, 25 to <29, and ≥ 30 years) and multiparous (yes/no). Models also included the following child variables as controls that related to BMI z-score trajectories from medical chart audits: birth weight (1^st^ quartile: <3265 grams, 2^nd^ and 3^rd^ quartile: 3265 - <3930 grams; and 4^th^ quartile ≥3930 grams); sex (female/male); attention defecit hyperactivity disorder (ADHD) medication use (yes/no); asthma medication use (yes/no); antidepressant medication use (yes/no); and the timing of the start of puberty from Tanner scoring (early, late, average).

#### Missing data and multiple imputations

Of the original sample of 595 children there were 517 with at least one BMI measurement after the age of 2 years. The baseline characteristics of the original population-based sample and the analysis sample were examined to see if variables of interest differed significantly (Table 1). None of the variables of interest differ significantly between the samples and thus the analysis sample remains a reasonable one to explore our hypotheses in the population-based birth cohort.

**Table 1:** Baseline characteristics of population-based sample and analysis sample

**Income measure**	**Population sample**		**Analysis sample**		**p-value**^**a**^
	**n = 595**		**n = 517**		
	**%**	**N**	**%**	**N**	
**Low-income at birth**					0.50
Yes	44	257	43	220	
No	56	334	57	293	
*Missing*		4		4	
**Early-life risk factors**					
**Maternal overweight/obesity**					0.57
Yes	50	295	49	254	
No	50	300	51	263	
**Gestational weight gain**					0.28
Excessive	47	282	47	244	
Inadequate	18	106	17	88	
Within recommendations	35	207	36	185	
**Smoking during pregnancy**					0.89
Yes	20	114	20	99	
No	80	477	80	414	
*Missing*		4		4	
**Control Variables**					
**Multiparious**					0.23
Yes	41	243	42	216	
No	49	352	48	301	
**Age category**					0.14
18 to 25 years	26	153	27	140	
25 years to 30 years	33	195	32	167	
Older than 30 years	41	247	41	210	
**Birthweight**					0.17
<3265 grams	26	157	25	128	
≥3930 grams	25	147	25	128	
3265 - <3930 grams	49	291	50	261	
**Sex**					0.12
Female	47	278	48	248	
Male	53	317	52	269	

^a^Chi-squared analysis p-value comparing analysis sample and those not included (n = 68) from the population sample.

The small amount of missing data on early-life risk factors and controls were imputed (Table 1). For example, smoking during pregnancy was missing for 4% of the sample. We used a fully conditional specification imputation method for categorical and discrete variables in PROC MI [41]. We used PROC LOGISTIC for the multivariate analysis and PROC MIANALYZE to pool the outcomes from the five imputed datasets.

None of the missing data for the trajectory analysis was imputed since the longitudinal latent class modeling uses all available data. We ran sensitivity analysis to determine if the frequency of the measures would bias the results. The complete case model used only children with at least one measure every 3 years. The full model used any child with measures regardless of the frequency. The family income trajectory classifications were not statistically different for the complete-case and full models. The complete-case model for BMI z-score trajectory had a higher proportion of obese, 14% v. 12%, and “become-overweight” children, 20% v 15%, when compared to the full model. Therefore, children with fewer measures are more likely to be classified as normal weight and make our findings conservative since they are the reference group in our analysis.

All data analysis for this paper was conducted using SAS® software (Version 9.3, 2012, SAS Institute, Cary, NC).

## Results

### BMI z-score trajectory

**Figure 1:**
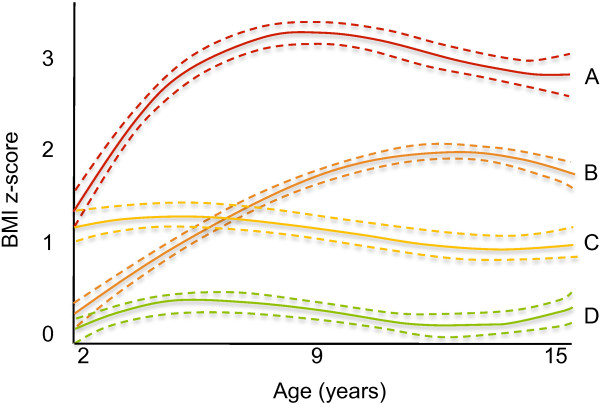
**Latent-class modeling of BMI z-score trajectories, 2 to 15 years (n = 517).** Legend: BMI z-score trajectories. – – – – – – 95% confidence interval. A “red line” Obese (12%). B “orange line” Become-overweight (15%). C “yellow line” Overweight-stable (20%). D “green line” Not-overweight (53%).

In the latent-class analysis for BMI z-score trajectory there was strong evidence based on the change BIC score with the addition of each additional class up to 6 classes (Additional file 5: Table S4). However, upon subsequent visual inspection it became apparent that this model had redundant groups from a public health perspective. For example, a stable trajectory with BMI z-score at 0 was very similar to a stable trajectory with BMI z-score at −0.5. In addition, a severely obese trajectory had only 11 subjects. Therefore, models with fewer classes were examined. The 4-class model best represented the expected and theoretically important trajectories capturing both stable and rising trajectories within the obese, overweight, and normal ranges. The 4 distinct BMI z-score trajectories identified were: *obese*, 12% of sample; *become-overweight*, 15%; *overweight-stable*, 20%; and *not overweight*, 53% (Figure 1).

### Family income trajectory

**Figure 2:**
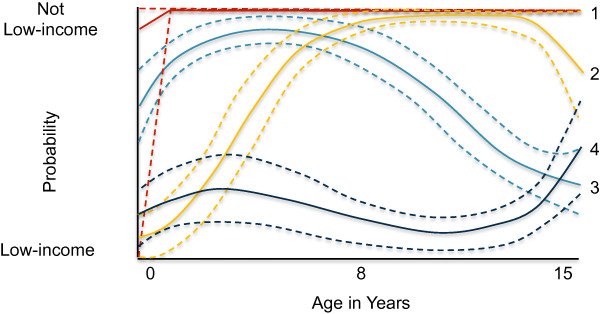
**Latent-class modeling of income trajectories, 0 to 15 years (n = 517).** Legend: Income trajectories. – – – – – – 95% confidence interval. 1 “red line” Not low-income (56%). 2 “yellow line” Upwardly-mobile (15%). 3 “light blue line” Downwardly-mobile (17)%. 4 “dark blue line” Persistently low-income (12%).

In the latent class analysis for family income trajectory, the 5-class model had the strongest evidence based on change in BIC (Additional file 6: Table S5). The 5-group model was very similar to the 4-group model, although it included an additional and earlier upwardly mobile group. The 4-group model was chosen as the final model because the additional class in the 5-group model was not theoretically different enough to justify including. The four income trajectories identified by the latent class analysis were *persistent low-income*, 12% of the sample; *downwardly-mobile*, 17%; *upwardly-mobile*, 15%; and *not low-income*, 56% (Figure 2).

### BMI z-score trajectory classification based on family income trajectory and early-life risk factors

We found that *persistent low-income* children were more likely to be in the *overweight-stable* trajectory (AOR = 2.55, 95% CI = 1.03, 5.42) and *downwardly-mobile* children were more likely to be in the *obese* trajectory (AOR = 2.36, 95% CI = 1.12, 5.77) compared to not-low-income children (Table 2). Interesting, *upwardly-mobile* children do not significantly differ in BMI z-score trajectory classification compared to *not-low-income* children.

**Table 2:** Odds ratios from multinomial logistic regression of family income trajectory and early-life risk factors for BMI z-score trajectory classification

	**Obese**				**Become-overweight**				**Overweight-stable**			
	**n = 69**				**n = 92**				**n = 119**			
	**Unadjusted**		**Adjusted**^**a**^		**Unadjusted**		**Adjusted**^**a**^		**Unadjusted**		**Adjusted**^**a**^	
	**OR**	**CI**	**OR**	**CI**	**OR**	**CI**	**OR**	**CI**	**OR**	**CI**	**OR**	**CI**
**Income trajectory**												
Persistent low-income	2.26	0.87, 5.88	1.38	0.44, 4.33	1.61	0.69, 3.88	1.33	0.53, 3.33	**3.00**	**1.51, 5.98**	**2.55**	**1.03, 5.42**
Downwardly-mobile	**2.99**	**1.51, 5.93**	**2.36**	**1.12, 5.77**	1.22	0.61, 2.43	1.04	0.50, 2.18	0.95	0.48, 1.90	0.82	0.38, 1.79
Upwardly-mobile	1.62	0.74, 3.52	1.15	0.46, 2.92	0.73	0.33, 1.61	0.59	0.25, 1.39	0.89	0.45, 1.77	0.68	0.31, 1.50
Not low-income	1.00	-	1.00	-	1.00	-	1.00	-	1.00	-	1.00	-
**Maternal overweight/obesity**												
Yes	**7.02**	**3.57, 13.77**	**8.31**	**3.80, 18.2**	**2.85**	**1.69, 4.78**	**2.37**	**1.34, 4.22**	**1.59**	**1.00, 2.51**	**1.79**	**1.02, 3.14**
No	1.00	-	1.00	-	1.00	-	1.00	-	1.00	-	1.00	-
**Gestational weight gain**												
Excessive	1.21	0.67, 2.19	0.49	0.23, 1.05	**2.37**	**1.28, 4.38**	**2.01**	**1.01, 4.00**	0.82	0.50, 1.35	0.61	0.33, 1.12
Inadequate	0.56	0.28, 1.40	0.58	0.21, 1.66	1.53	0.70, 3.33	1.47	0.64, 3.37	0.54	0.27, 1.08	0.68	0.31, 1.50
Adequate	1.00	-	1.00	-	1.00	-	1.00	-	1.00	-	1.00	-
**Early-life rapid weight gain trajectory**												
High-rising	**7.03**	**3.05, 16.22**	**5.42**	**2.18, 13.44**	1.71	0.84, 3.49	1.64	0.77, 3.49	**3.32**	**1.85, 5.96**	**3.87**	**2.07, 7.26**
Low-rising	0.77	0.31, 1.95	**0.50**	**0.19, 1.36**	1.00	0.54, 1.89	0.90	0.46, 1.77	**0.29**	**0.14, 0.58**	**0.30**	**0.15, 0.63**
Stable	1.00	-	1.00	-	1.00	-	1.00	-	1.00	-	1.00	-
**Smoking during pregnancy**^**b**^												
Yes	**2.18**	**1.18, 4.03**	-	-	0.84	0.42, 1.67	-	-	1.37	0.78, 2.39	-	-
No	1.00	-	1.00	-	1.00	-	1.00	-	1.00	-	1.00	-
**Breastfeeding duration**^**b**^												
< 2 months	1.59	0.79, 3.20	-	-	1.09	0.59, 2.04	-	-	1.04	0.59, 1.84	-	-
2 to < 4 months	1.23	0.48, 3.12	-	-	1.19	0.54, 2.64	-	-	0.82	0.38, 1.80	-	-
4 to < 8 months	0.64	0.25, 1.63	-	-	0.64	0.29, 1.41	-	-	0.81	0.41, 1.57	-	-
≥ 8 months	1.00	-	1.00	-	1.00	-	1.00	-	1.00	-	1.00	-

Reference category for BMI z-score trajectory is not-overweight (n = 315).

^a^Mulitnomial logistic regression odds ratios are adjusted for multiparious, maternal age, sex of child, birth weight quartile, asthma medication use, ADD medicaiton use, antidepressant medication use, puberty status.

^b^Smoking during pregnancy was removed from the multivariate model because it was completely mediated by early-life rapid weight gain trajectory; breastfeeding was removed because it was not associated with BMI trajectories.

Bold denotes 95% CI for odds ratios that do not cross 1.

After controlling for family income trajectory, three early-life risk factors remained statistically significant in the final models: maternal overweight/obesity, excessive gestational weight gain, and child’s early-life weight gain trajectory. Maternal overweight/obesity was significantly associated with a child being in the *obese* (AOR = 8.31, 95% CI = 3.80, 18.20), *become-overweight* (AOR = 2.37, 95% CI = 1.34, 4.22), and *overweight-stable* trajectories (AOR = 1.79, 95% CI = 1.02, 3.14). Excessive gestational weight gain was associated with increased likelihood of a child being in the *become-overweight* trajectory (AOR = 2.01, 95% CI = 1.01, 4.00). Finally, both high-rising and low-rising early-life weight gain trajectories were associated with BMI z-score trajectory. The effect of smoking during pregnancy on BMI trajectory was completely mediated by birth weight and early-life weight gain trajectory. Breastfeeding was not significantly associated with BMI trajectory in the unadjusted model and therefore not included in the adjusted model.

## Discussion

This study was undertaken to assess the association between family income trajectory, early-life risk factors, and BMI z-score trajectory from birth to adolescence. The use of family income trajectory and BMI trajectories was unique and offers a lifecourse perspective on the relationship between family income and the development of overweight and obesity. Our main finding was that children who were persistently low-income are more likely to have a stable BMI z-score in the overweight range, while children who moved into low-income are more likely to develop a BMI z-score in the obese range.

Interestingly, we found that children who moved out of low-income are as likely to be overweight or obese compared to children who did not experience low-income. This finding was contrary to previous studies [9-17] that have found a strong correlation between a single measure of low-income in early childhood and obesity in later life. There are two potential explanations for this finding. First, the use of a longitudinal measure for income allowed us to disentangle “persistent low-income” children from “upwardly mobile” children and captured their different experiences. Alternatively, the effects of early childhood low-income may not have appeared in this cohort by the age 15.

The mechanisms that could produce overweight and obesity in children who move into low-income compared to children who were persistently low-income are likely different. We propose that the life events that cause a family to move into low-income, such as divorce or loss of a job, may put these children in stressful environments. Other researchers have highlighted the link between income and health across the lifespan being differential access to healthcare, environmental exposures, health behavior, and differential exposure to stress [42]. In the future, it would be helpful to know how the nutrition and physical activity habits of families change during times of economic transition.

The findings of the current study have important policy implications. More immediately, assistance programs need to support and encourage healthy eating and physical activity during times of economic downturn. A more upstream approach would be to support and encourage opportunities for education and training that would allow many economically disadvantaged parents to have upward social mobility and ultimately improve their children’s health [43].

In addition, our findings support the growing evidence that early-life risk factors are strongly associated with the development of overweight and obesity in children. Two other studies [22,23] have examined childhood BMI trajectories in association with early-life risk factors. In this study we explored five early-life risk factors in the context of a multidimensional measure of family income. Our findings that maternal overweight/obesity, excessive gestational weight gain and high-rising early-life weight gain trajectories were significantly associated with BMI trajectory support the findings of the previous two studies. These two studies did not find or report significant associations between BMI trajectory and a measure of SES [22,23]. Our findings suggest that obesity prevention needs to start early in the life cycle, for example during pregnancy. Efforts to prevent childhood obesity should be linked with efforts to reduce obesity in childbearing women and the prevention of excessive gestational weight gain.

Our study has five methodological strengths. First, we used an understudied rural population. The sample had considerably higher rates of overweight and obesity compared to the two other studies [22,23] that have looked at BMI trajectories. In a healthy population, we expect approximately 15% of children to be classified as overweight and 5% as obese. In this sample, 35% of children are grouped in an overweight trajectory and 12% are grouped in the obese trajectory. The average rates of overweight and obesity in New York State are around 40% [44]. The higher-than-expected proportion of overweight and obesity in the sample suggests that this population has unhealthy rates of weight gain.

Second, we examined the development of overweight and obesity from infancy to adolescence while most studies do not have data spanning all of childhood. Third, there was relatively low attrition for a longitudinal study of this nature. The free school-based clinics and a multi-site healthcare system mitigate against attrition and facilitate the retention of low-income children. The loss to follow-up was therefore reduced and the maintenance of more low-income adolescents in the study reduces bias. Low-income children were not significantly more likely to have less than median BMI measures compared to not low-income children (49% v. 45%, respectively, chi-square p-value = 0.333). Fourth, we used only measured weights and heights to define BMI z-score trajectories over time, of which 88% were from pediatric well-child visits. Fifth, we used group-based trajectory analysis based on latent class modeling to create income and BMI z-score trajectories. This allowed us to explore trends that emerged across all of childhood and efficiently and prudently categorize children using data form multiple time points.

Our findings should be considered in the context of their limitations. First, the measure of low-income was a dichotomous measure of income and may mask important differences within the two broad income groups. The use of insurance codes to measure income was a unique aspect of this study. Insurance codes were recorded for billing purposes at the time of the doctor visit and require thorough income documentation to qualify for Medicaid or Child Health Plus insurance. This makes insurance code a more objective measure of low-income across time compared to self-reported income.

Second, we only have measures from the time points when children attended a clinic or hospital. Third, while our sample was predominantly white and rural our findings corroborate previous work done by Kendzor et al. [20] in a more diverse and representative sample. Kendzor et al. used data from the Study of Early Child Care and Youth Development between 1991 and 2007 at 10 sites across the United States [20]. Like us, they found that children with downwardly mobile and stable low-incomes had higher adiposity at age 15. In addition, rural populations, which are predominantly white, are often understudied even though they comprise 15% of the US population and experience higher rates of child poverty compared to metropolitan areas [45]. Finally, we used a discrete approximation of a complex, continuous situation and the trajectories are an estimate. Future research is needed to identify income and BMI trajectories using a nationally representative sample to examine their associations.

## Conclusion

These findings demonstrate that overweight and obesity develops across childhood with distinguishable BMI z-score trajectories occurring in early childhood and carrying on through adolescence. This work further supports the growing evidence that there are several preventable early-life obesity risk factors that could be targeted for intervention. In addition, income trajectories are independently associated with these early-life obesity risk factors and the development of overweight and obesity across childhood. Therefore, a holistic approach that considers family income and risk factors will be critical for the development of policies and interventions for preventing overweight and obesity in children.

## Consent

Written informed consent was obtained from the patient for the publication of this report and any accompanying images.

## Competing interests

The authors declare that they have no competing interests.

## Authors’ contributions

The authors’ responsibilities were as follows--MMD, JDH, CMO designed research; MMD conducted research; MMD analyzed data; MMD and CMO wrote the paper; MMD had primary responsibility for final content. All authors read and approved the final manuscript.

## Pre-publication history

The pre-publication history for this paper can be accessed here:

http://www.biomedcentral.com/1471-2458/14/417/prepub

## Supplementary Material

Additional file 1: Table S1 Mixture fit statistics for breastfeeding duration trajectory models. Description: Table S1 provides mixture fit statistics for trajectory models. Click here for file

Additional file 2: Table S2 Mixture fit statistics for early-life rapid weight gain trajectory models. Description: Table S2 provides mixture fit statistics for trajectory models. Click here for file

Additional file 3: Figure S1 Latent-class modeling of weight-for-length z-score trajectories, 0 to 2 years (n = 517). Description: Figure S1 illustrates the latent-class modeling of weight-for-length z-score trajectories from 0 to 24 months. Click here for file

Additional file 4: Table S3 Chi-squared analysis of income trajectory and early-life risk factors. Description: Table S3 provides information on the associations between income trajectory and early-life obesity risk factors. Click here for file

Additional file 5: Table S4 Mixture fit statistics for BMI z-score trajectory models. Description: Table S4 provides mixture fit statistics for trajectory models. Click here for file

Additional file 6: Table S5 Mixture fit statistics for family income trajectory models. Description: Table S5 provides mixture fit statistics for trajectory models. Click here for file
