# Supplementary material for: Changes in family income status and the development of overweight and obesity from 2 to 15 years: a longitudinal study
Source: BMC Public Health. 2014 May 1;14:417. doi: 10.1186/1471-2458-14-417 (PMC4041137; doi:10.1186/1471-2458-14-417)
Supplement: Additional file 3: Figure S1 — Latent-class modeling of weight-for-length z-score trajectories, 0 to 2 years (n = 517). Description: Figure S1 illustrates the latent-class modeling of weight-for-length z-score trajectories from 0 to 24 months. [file 1471-2458-14-417-S3.pdf]

Additional Figure 1. Latent-class modeling of weight-for-length z-score trajectories, 0 to 2 years (n=517)

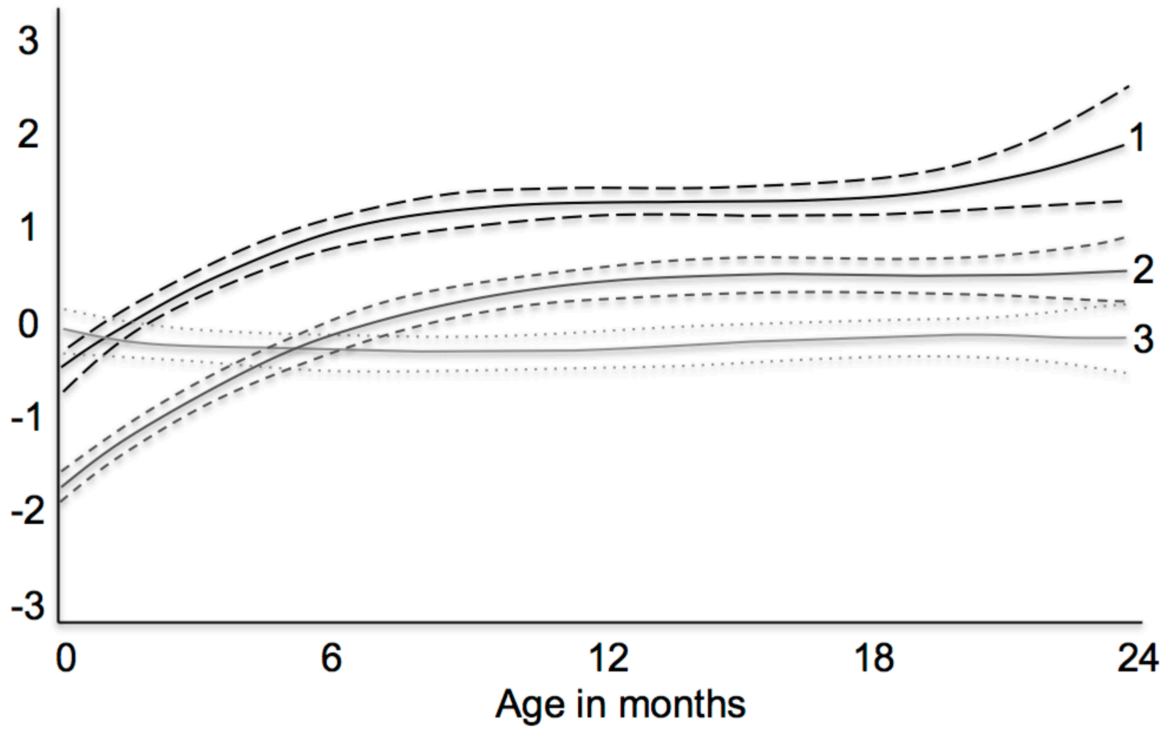

Legend for Supplemental Figure 1

Early-life rapid weight gain trajectories

- - - - 95% confidence interval
1 ▬ High-rapid weight gain (35%)
2 ▬ Low-rapid weight gain (42%)
3 ▬ Stable (23%)
